# Comparing the effects of low-dose contraceptive pills to control dysfunctional uterine bleeding by oral and vaginal methods

**DOI:** 10.12669/pjms.295.4013

**Published:** 2013

**Authors:** Ferdous Mehrabian, Fariba Abbassi

**Affiliations:** 1 Ferdous Mehrabian, MD, Associate Professor of Obstetrics & Gynecology, Obstetrics & Gynecology Department, Faculty of Medicine, Isfahan University of Medical Sciences, Isfahan, Iran.; 2 Fariba Abbassi, MD, Physician, Obstetrics & Gynecology Department, Faculty of Medicine, Isfahan University of Medical Sciences, Isfahan, Iran.

**Keywords:** Contraceptive pill, vaginal administration, Dysfunctional uterine bleeding

## Abstract

***Background and*** ***Objective*** ***:*** Contraceptive pills are generally taken orally and can cause side effects such as nausea, vomiting and hypertension. The vaginal use of these pills can reduce such complications. Our objective was to compare the efficacy and side effects of low dose contraceptive pills by oral and vaginal route in the management of dysfunctional uterine bleeding-(DUB)

***Methods:*** This comparative observational study was conducted at Beheshti and Alzahra (SA) teaching hospitals, affiliated to Isfahan University of Medical Sciences in 2010-2011. One hundred women who presented with DUB were randomly assigned into two groups of equal number, receiving the low dose oral contraceptive pills by oral or vaginal route for three month. The amount and duration of bleeding were compared at the beginning and at the end of the study and side effects by these two methods compared.

***Results: ***The results of this study showed that both oral and vaginal routes effectively reduced the duration and amount of bleeding due to DUB after three courses of treatment. This effect was better in the vaginal method compared with oral administration (P = 0.03). Regarding the side effects, nausea and vomiting were significantly higher in the oral group than in the vaginal group (P = 0.03). Vulvovaginitis infection was more frequent in the vaginal group than in the oral group (P = 0.03).

***Conclusion:*** Low dose contraceptive pills are effective in reducing the amount, time, and duration of bleeding in patients with DUB. In addition, reduction of gastrointestinal side effects by vaginal route helps to use these pills by the patient with proper training of physicians, midwives and patients.

## INTRODUCTION

Dysfunctional uterine bleeding is defined as abnormal uterine bleeding in the absence of organic disease.^1^ The diagnosis can only be made after all other causes for abnormal or heavy uterine bleeding have been excluded. The pathophysiology is largely unknown.^2^ One of the effective methods in the treatment of prolonged bleedings is the use of contraceptives, which in addition to disease control, reduces the likelihood of pregnancy in these patients.^2^ Despite the suitable effectiveness of these pills in the treatment of these diseases, the related side effects of these pills such as vomiting, headache, mood changes, pills adoption, the risk of hypertension and thrombo-embolic events are a major problem.^3^

In recent years, different routes of using contraceptives have been considered and attention has been drawn to use them vaginally. In the vaginal method, intestinal - gastric medicine absorption and hepatic first-pass effect are eliminated. Therefore, the side effects decrease substantially.^3^^-^^5^ Drug absorption is slower in vaginal medication resulting in lower blood concentrations, however the bioavailability is equal to the oral method.^6^ Vaginal administration of steroids contraceptive reduces the mentioned side effects. In this method, steroid contraceptives are gradually absorbed into the vaginal mucosa and get directly into the bloodstream. As a result, there are fewer gastrointestinal adverse and systemic effects compared with OCP.^7^

Several studies have been conducted in the case of the administration of low-dose contraceptive pills through the vagina in order to prevent pregnancy, comparing its performance and side effects with oral medications. Based on these studies, the vaginal use of these medicines has been effective in the suppression of ovulation.^5^^,^^8^ Data regarding use of contraceptive pills by vaginal route in the management of DUB is scarce. The objective of the present study was to assess the efficacy and tolerability of these pills in reducing the amount and time of DUB by oral and vaginal use.

## METHODS

This comparative observational study was conducted at Beheshti and Alzahra (SA) teaching hospitals, affiliated to Isfahan University of Medical Sciences in 2010-2011. All women with vaginal bleeding of unknown etiology who were referred to these centers were evaluated. All patients were married. Detailed history was taken and complete physical examination was carried out, followed by pelvic examination. Patients with normal pelvic examination underwent sonographic examination. Only patients with endometrial thickness less than 5 mm were enrolled in this study.

The inclusion criteria consisted of :1 - Normal pelvic examination; 2 - Endometrial thickness less than 5 mm in ultrasound immediately after bleeding; 3 - Normal Pap smear tests; 4 - Negative pregnancy test (Beta HCG); 5 - No absolute contra indications for using the pills.

Absolute contra indications of using oral contraceptives with low-dose (LD OCP) were considered as:1 – Thrombus-embolic probability; 2 - Significant impairment of liver function; 3 - Known or suspected breast cancer; 4 - Known or suspected pregnancy; 5 - Smokers over 35 years;6 - Migraine headaches;7 - High blood pressure; 8 - The decision to have elective surgery; and/or 9 - Genital infection risk factors (diabetes, chronic use of corticosteroids, immunodeficiency disease including HIV, history of treated infection or vaginal symptoms in less than one year).

Eligible women were divided into two groups randomly. In one group, the low-dose (LD) pills were prescribed orally for three months. The second group received the same drug through vaginal route. Patients were followed up for three months. In the event of severe bleeding in these patients during the study, the treatment was initiated by injection of estrogen and progestin. Afterwards, the conservative treatment was continued by using LD OCP orally or vaginally. A questionnaire was used in order to measure the amount of bleeding and its eventual decline following the completion of the study. The amount of bleeding was measured by using the pictorial bleeding assessment chart. Each chart, as an image was given to the patients and had five figures. (a) one pad, (b) ¾ pad, (c) ½ pad, (d) ¼ pad and (e) 1/5 pad. At the end of the study, the patients were asked again to complete the above chart to determine the amount of bleeding. Additionally, the bleeding time was also recorded at both the start and the end of the study. The questionnaire included information about the effects of drug use such as gastrointestinal side effects, skin problems, mood disturbances, headache, blood pressure, spotting between periods and vulvovaginitis.

## RESULTS

The mean age of the women in the group with the consumption of LD pills vaginally was 43.2 ± 8.4 years and it was 40.28 ± 7.08 years in the oral group.(p = 0.068 In the vaginal group, before starting the study, the mean consumption of pad was 19.8 ± 4.2 and it was 18.8 ± 4.6 in the oral group. (p = 0.057), there was no significant difference between the two groups. After the end of the study, in vaginal group, this amount was reduced to 12.9 ± 4.5 and in the oral group; it was reduced to 12.1 ± 2.6. (p = 0.29), with no significant difference. The mean duration of bleeding in vaginal group was 9.7 ± 1.9 days and 9.6 ± 1.9 days in oral group before starting the study. At baseline, during bleeding and after the end of the study, this period was reduced to 7.6 ± 2.1 days in oral group and to 6.72 ± 1.2 days in vaginal group. (p = 0.01), the difference between the two groups was significant. (Table-I)

**Table-I:** Comparison of the age, the amount of bleeding and bleeding time in the two groups studied

*Variables*	*Vaginal group*		*Oral group*		*P-value*
	*Mean ±*	*SD*	*Mean ±*	*SD*	
Age	43.2 ±	8.4	40.28 ±	7.08	0.064
*Consumption of pads*					
before the study	19.8 ±	4.2	18.8 ±	4.6	0.057
after the study	12.9 ±	4.5	12.1 ±	2.6	0.29
*Duration of Bleeding(days)*					
before the study	9.7 ±	1.9	9.6 ±	1.9	0.93
after the study	6.72 ±	1.2	7.6 ±	2.1	0.01

**Table-II:** Comparison of Side Effects between two groups

Variables	Oral route	Vaginal route	p-value
	(n)	(n)	
Nausea and vomiting	7	1	0.03
Acne	1	-	0.05
Headache	6	1	0.056
Mood changes	7	-	0.05
Spotting	10	4	0.074
Vulvovaginitis	5	13	0.033

The incidence of side effects was compared between the groups. Among the 100 studied patients in this study, eight patients had gastrointestinal complications such as nausea and vomiting. Among them, there were seven patients in the LD oral group and one patient in the vaginal group. Skin problems an increase in acne was reported in only one patient who was in the oral group. 

Headache was reported in seven patients, one subject was in vaginal group and six patients were in the oral group. Although, there were more patients with headache in the oral group, this difference was not significant between the two groups (p = 0.056). Mood changes (such as depression, irritability and anger) were reported by seven patients in the oral group. Spotting in between periods was reported in 14 patients four in the vaginal group and 10 patients in the oral group. Although, the number of spotting cases in the oral group was more than the vaginal group, this difference was not significant (p = 0.074). Most of spotting cases were in the vaginal group and in the middle of the period (3 cases). The oral group frequently had spotting in two or three days before the start of menstruation. Symptoms of Vulvovaginitis (itching, burning and discharge) were reported in 18 patients out of which 13 patients were in the vaginal group. (p = 0.033), the difference between the two groups was highly significant. (Table-II).

## DISCUSSION

This study compared the efficacy and side effects of low-dose contraceptives used either orally (OCP) or vaginally (VCP). The study investigated the effect of these two methods in reducing the amount and duration of bleeding in women with dysfunctional uterine bleeding. The results of the present study showed that both methods could effectively reduce the amount of bleeding among the patients.

Vaginal absorption of released steroids from CVR is gradual and reaches to a relatively constant level in the circulatory system. Despite the fact that the blood levels of steroids in this method is low there is maximum level of hormone in the use of oral steroids for an effective contraception. The rate of pregnancy is one to two per 100 women per year. Therefore, there is no difference in pregnancy rate.^9^ However, in the reviewed studies, none of them had studied directly about the effect of vaginal pills to reduce bleeding. In the present study, the amount of bleeding was reduced to a considerable amount in both groups and the duration of vaginal bleeding was reduced significantly with vaginal route than the oral method due to the gradual increase of hormone levels in the blood stream and reaching to steady blood levels. Thus, the slower absorption of drugs through the vagina results in achieving blood levels of progesterone to a constant concentration. This will lead to a better stability of the endometrium and finally, less blood loss.^10^ The overall obtained results of the study showed that the use of vaginal LD pills are more effective than the oral administration and reduces the bleeding time, substantially.

Results of gastrointestinal side effects were similar to other studies as regards the side effects of vaginal LD pills compared to oral administration. The results also showed that gastrointestinal side effects of contraceptive low-dose (LD) drugs in vaginal use, is substantially less than oral administration.^11^ This is due to the direct contact of the drug with gastrointestinal wall and the elimination of hepatic first pass. The mood effects of using LD contraceptives in this study were observed in 14% patients among the users of oral medication. However, other studies have reported the frequency of mood changes in 7% of patients.^2^^,^^12^^,^^13^ This two-fold increase in the incidence of this disorder could be due to the patients' lack of familiarity, definition and assessment of these disorders. It should be noted that mood disorder was not observed in patients who were taking the medicine vaginally, which is a positive and significant point.

In using the vaginal LD pills, the results of the present study showed that spotting cases and break through bleeding (BTB) were less in oral group; however this difference was not significant. The obtained results are consistent with some other studies.^12^^-^^14^ It was also observed in these studies that most of spotting and BTB occurred in oral administration group. In our study we have detected that most BTB cases in the vaginal group occurred in the middle of the period (3 out of 4 cases). However, the spotting in oral administration occurred two to three days before the onset of menses.

The frequencies of vulvo-vaginitis among the patients with LD pills in vaginal method were 72.2%. This could be due to the failure to observe proper hygiene by women during insertion of vaginal pills. To resolve this difficulty, there is a need for patient education.^15^ These results suggest that it is necessary to conduct studies about the effect of vaginal LD on the vaginal flora and it should be investigated that whether LD pills in vaginal method can upset the normal flora on the vagina and thus predispose the patients to vaginal infection or not?

## CONCLUSION

Low dose contraceptive pills by vaginal route can be used as effectively as oral methods in controlling the amount and duration of bleeding in patients with DUB with fewer side effects,

## Notes

***Acknowledgment:*** This study was conducted as a Thesis funded by Isfahan University of Medical Sciences.
